# Health Domains for Sale: The Need for Global Health Internet Governance

**DOI:** 10.2196/jmir.3276

**Published:** 2014-03-05

**Authors:** Tim Ken Mackey, Bryan A Liang, Jillian C Kohler, Amir Attaran

**Affiliations:** ^1^Department of AnethesiologyUniversity of California, San DiegoSan Diego, CAUnited States; ^2^Leslie Dan Faculty of PharmacyUniversity of TorontoToronto, ONCanada; ^3^Faculty of LawUniversity of OttawaOttawa, ONCanada

**Keywords:** eHealth, global health governance, information technology, Internet, domain names

## Abstract

A debate on Internet governance for health, or “eHealth governance”, is emerging with the impending award of a new dot-health (.health) generic top-level domain name (gTLD) along with a host of other health-related domains. This development is critical as it will shape the future of the health Internet, allowing largely unrestricted use of .health second-level domain names by future registrants, raising concerns about the potential for privacy, use and marketing of health-related information, credibility of online health content, and potential for Internet fraud and abuse. Yet, prospective .health gTLD applicants do not provide adequate safeguards for use of .health or related domains and have few or no ties to the global health community. If approved, one of these for-profit corporate applicants would effectively control the future of the .health address on the Internet with arguably no active oversight from important international public health stakeholders. This would represent a lost opportunity for the public health, medical, and broader health community in establishing a trusted, transparent and reliable source for health on the Internet. Countries, medical associations, civil society, and consumer advocates have objected to these applications on grounds that they do not meet the public interest. We argue that there is an immediate need for action to postpone awarding of the .health gTLD and other health-related gTLDs to address these concerns and ensure the appropriate development of sound eHealth governance rules, principles, and use. This would support the crucial need of ensuring access to quality and evidence-based sources of health information online, as well as establishing a safe and reliable space on the Internet for health. We believe, if properly governed, .health and other domains could represent such a promise in the future.

## Background

A debate on Internet governance for health, or “eHealth governance”, is emerging with the impending award of a number of new health-related “generic top-level-domain names” (gTLDs; eg, similar to .edu for educational institutions) that could shape the future of online health information. The Internet, which consists of a hierarchical domain naming system of IP addresses of computers, services, and other digital resources, relies on domain names as an easily recognizable way for users to search and navigate online content. The Internet Corporation for Assigned Names and Numbers (ICANN), a nonprofit corporation founded in 1998 that controls this system, is currently undergoing the largest expansion of the Internet in history [1]. It is adding over a thousand new gTLDs, potentially including a new .health domain and close to 20 other gTLDs related to medicine and health, which are scheduled to become active as early as the beginning of 2014. Yet, ICANN’s complex and highly political process of awarding health-related gTLDs could have a profound impact on information privacy, use, and sale; health marketing; and content quality that could influence future trust, security, and credibility of the health Internet. Hence, it is critical that applicants are carefully scrutinized to ensure that they are abiding by ethical principles, practices, and rules with respect to public health and the public interest.

Despite this need for careful consideration, it appears that current applicants for health-related gTLDs are highly varied, with few having strong ties to the global medical or public health community. Indeed, there are significant doubts of whether they will meet the needs of the broader public interest. Below, we describe the current debate surrounding the new .health domain as well as provide an overview of other health-related gTLDs within the context of the growing importance of the Internet on health behavior and information-seeking. We argue that there is a crucial need for better governance to enable evidence-based sources and to ensure .health and other domains represent a safe space on the Internet for health, rather than simply an unregulated space for health marketing.

## Health Information and the Internet

Current controversy surrounding health domains is rooted in the Internet’s growing importance as a health information source. In 2013, the International Telecommunication Union estimated that 38.8% (2.7 billion) of the world’s population used the Internet [2]. Many of these users are seeking important health information online [2,3]. In the United States, surveys report 72% of online adults accessed the Internet to find health information primarily on the subjects of diseases and treatments [4]. Other regions, including the European Union and emerging markets, have also shown marked increases in online health information seeking and self-diagnosing behavior [3,5,6].

The promise of accurate and reliable health information online is its potential to empower patient participation and inform decision making. Yet there are risks beyond inaccurate information online, including consumer privacy issues, false, or misleading promotion directed towards health consumers/products, ensuring appropriate regulation of commercial health marketing, and cybersecurity-related health issues (such as health-related spam and financial fraud). These risks underlie the need to ensure a trusted online health environment that promotes consumer empowerment and public health [3]. But these risks are further exacerbated by a lack of global Internet governance. The result has been the proliferation of numerous types of health-related information sites without content quality assessment [3]. With more than 100,000 health-related websites estimated to be in existence, Internet users may have difficulty accessing evidence-based sources and often seek information through simple search engine (eg, Google, Yahoo, Bing) queries that may prioritize sites of lower quality, undisclosed commercially sponsored content, irrelevant information, and/or at worst, misinformation [3,7]. For example, illicit online pharmacies have been detected illegally marketing and selling pharmaceuticals without a prescription, misrepresenting crucial risk information, and not disclosing other risks of their often counterfeit and otherwise dangerous products [7-9].

In response, initiatives to direct users to medically dedicated online health information sources have been explored. This includes deployment of medical search engines (eg, Healthfinder), voluntary certification programs for online content (eg, HealthOntheNetFoundation Code/search toolbar), and websites backed by a well-known public or private health care delivery sources (eg, UK National Health Service). Yet, low user adoption, health literacy issues, and the growing popularity of alternative sources for health information (including social media) may pose ongoing challenges for future success of these efforts [3,7]. Hence, having convenient access to a safe and reliable source of health content online remains a critical concern for billions of global users increasingly relying on the Internet for health.

## Expansion of the Internet

### ICANN’s New Naming Program

In June 2008, ICANN began the creation of a new program of expanding naming of the Internet from the original two limited rounds of applications for new top-level-domain (TLD) names conducted in 2000 (proof-of-concept round) and 2004. This new program opened up in 2011, allowing for the creation of numerous specific new gTLDs, expanding from the original list of 22 TLDs then in existence and aimed at vastly expanding the Internet name space. This expansion specifically included new unsponsored gTLDs (ie, operated under standard policies of ICANN’s processes) that generally consist of three or more characters and are open for any purpose or use.

ICANN’s new naming program allowed proposals for virtually any new domain name *suggested by applicants*, including those in different languages/characters, comprising numbers, and even using company brand names [10]. Acceptance of applications for new gTLDs began in January 2012 and has led to submission of nearly 2000 applications for a wide variety of gTLD strings [1]. Included were gTLDs that are geographic (eg, .paris, .Africa), general term domains (eg, .law, .money, .science, .sex), and those for specific entities (eg, .mit for Massachusetts Institute of Technology, .apple for Apple Inc, .bbc for the British Broadcasting Corporation, .McDonalds for McDonald’s Corporation). ICANN anticipated the first group of new gTLDs passing through the application process to be operational in late 2013 [11]. However, final award of new gTLDs will undergo a complex evaluation process that could include considerable costly delays and also potentially end in award of gTLDs through an auction process (with the highest bidder being awarded a gTLD) when there are several contending applicants. This procedural complexity has led to intense lobbying pressure on ICANN to allow applications to proceed despite documented objections from countries, international organizations, consumer advocacy groups, independent watchdogs, and the broader public community. Further, this process would ostensibly favor richer and more well-connected applicants—a system that many observers find questionable and lacking credibility.

Importantly, included in this new program were a number of proposals by various entities for a new .health domain and other health-related gTLDs. Applications are predominantly from private corporate entities with most having little or no history in medicine nor public health, include large pharmaceutical manufacturers applying for gTLD proprietary/trademark names, and comprise only a handful from health organizations and associations. This uneven mix of applicants creates uncertainty for the future prospects of a trusted space for online health content, which we describe below.

### .health Domain Applicants

The domain central in the current debate is the proposed “.health” gTLD [1]. Yet recognition of a need to create a dedicated Internet space for health is not new. More than a decade ago, the need for a trusted .health TLD with collaboration and oversight by the international community was explored [3,12]. In 2000, shortly after the creation of ICANN, the World Health Organization (WHO) and other stakeholders proposed formation of a .health TLD, but ultimately its proposal was not chosen as one of the seven proof-of-concept names for new TLDs during that round [12,13]. Subsequently, the new ICANN naming program announced in 2008 provided another opportunity to revisit the creation of .health (including possible resubmission of the original WHO proposal). Yet since this time, WHO and other public health-related stakeholders have been conspicuously absent in actively seeking the domain.

Instead, the current round of .health applicants consists exclusively of for-profit, private sector entities, all of which passed ICANN’s initial assessment and remain unopposed (Table 1), meaning they have the potential to be awarded the gTLD through ICANN’s evaluation process that will most likely end in an auction to the highest bidder. All are in single legal jurisdictions (eg, not represented by nor coordinating with any international/global health organization) and hence lack global geographic scope and representation. Indeed, simply on the basis of corporate law, none of the for-profit applicants are even publicly traded companies. This brings into question levels of transparency/disclosure, sufficiency of corporate governance (eg, those required under the US Sarbanes-Oxley Act and similar iterations in Australia, Canada, France, Germany, India, Japan, The Netherlands, South Africa, and other countries), ongoing financial viability, and accountability to the public.

**Table 1:** health gTLD applicants.

Applicant name	Application type, country, and status	Entity type	Affiliations	Proposed governance and criteria	Health sector support/ partnerships
DotHealth, LLC	Standard open gTLD USA Unchallenged; IO objection denied; award likely subject to auction.	Limited liability company	DotHealth, LLC (self)	Partnership with Neustar and Legitscript but no other formal governance structure. Purported use of policies, safeguards, and standard operating procedures to be actively monitored and enforced for inaccurate or misleading information (including illicit online pharmacies) in conjunction with partners. Also states that it will protect the name of the WHO’s second-level domain names within health TLD (public interest commitments).	National Association of Boards of Pharmacy; Inter-American College of Physicians and Services (website not functional); Association of Black Cardiologists; World Federation of Chiropractic; Regulatory Harmonization Institute
Afilias Limited	Standard open gTLD Ireland Unchallenged; IO objection denied; award likely subject to auction.	Irish company limited by shares	Afilias Limited (self)	None specifically listed. Persons or entities licensed as a health care provider (public interest commitments).	None listed
Goose Fest, LLC	Standard open gTLD USA Unchallenged; IO objection denied; award likely subject to auction.	Limited liability company	Donuts Inc (parent applicant) Parent company: Covered TLD, LLC	None specifically listed. Generally open entry with certain security/abuse prevention mechanisms in place.	None listed; mentions on website that company has substantial funding from private equity and venture capital funds
dot Health Limited	Standard open gTLD Gibraltar Withdrawn	Limited liability company	CEO – Famous Four Media Limited Parent company: Domain Venture Partners PCC Limited	Establishment of Governance Council consisting of key sector stakeholders that self nominate to participate. Generally open entry with certain security/abuse prevention mechanisms in place. Will implement additional protections for IGOs for second-level domain names.	None listed

Further, no applicants are familiar to the health field, but rather include companies such as various affiliates of Donuts Inc, which is attempting to obtain not just .health but has applied for more than 300 gTLDs under a number of different subsidiaries, most removed from health (eg, .apartments, .beauty, .casino, .dating, and even .wtf), suggesting the company is not focused on the prudent stewardship of patient-centered health information. Indeed, Donuts Inc is primarily backed by private equity/venture capital funds that has invested some $57 million in an attempt to secure gTLDs—a move that has raised concern among industry and Internet watchdogs [14]. Alarmingly, it has also been reported as being connected with other Internet companies that have provided services to spammers and cybersquatters, raising concerns about potential Internet fraud and abuse if Donuts is awarded its applied-for gTLDs [14].

Furthermore, of the current .health applicants, there are no developing countries, international/intergovernmental organizations, nonprofits, foundations, nor civil society groups as primary applicants. Only one applicant, DotHealth LLC, provides any support letters or specific inclusion from health-related stakeholders. However, DotHealth LLC supporters clearly do not constitute an adequate representation of the global health community. Indeed, all DotHealth LLC (only recently formed in 2011 based on its incorporation documents) supporters are US-focused, several are recently formed entities, one supporter’s website is non-functional, and many have close ties to industry.

Absence of active participation by public health stakeholders may point to a lack of attention and priority setting in recognizing the potential importance of a .health domain. Further, public health actors may lack technical expertise and financial resources to submit a viable application, similar to concerns raised in the original WHO ICANN proposal [3,12]. ICANN’s listed high entry and maintenance cost of gTLDs (including initial application fee of US $185,000 and annual fee of US $25,000) are not prohibitive for businesses generating profits but may be difficult to afford for the vast majority of global health organizations relying on unstable funding [1,3,10].

It is also unclear how current .health applicants would manage potential registrant quality and trustworthiness for .health use, or, in a related issue, possible categorization of restricted/reserved .health domain names. Since the applicants themselves are commercial, their ICANN .health applications indicate few if any restrictions on future .health registrants and would mostly offer registration and sale of .health use broadly. Only one of the applicants, Afilias Limited, indicates that under its revised criteria in response to public interest concerns, .health registrants would have to at least hold a health care provider license. Yet, it should be noted that requirement for licensure verification does not guarantee any level of protection for consumers, does not guarantee appropriate oversight, and is not sufficient in itself to safeguard the public interest.

Some hypothetical examples of .health gTLD second-level domain names that could be misused are provided in Table 2 and illustrate the immediate risk of unrestricted use proposed by the majority of current .health applicants. Indeed, in certain circumstances where there are multiple .health second-level domain registrants, award may be conducted by a third-party auction/broker system to the highest bidder, which would ostensibly favor for-profit or wealthier entities/individuals.

**Table 2:** Examples of potential misuse of .health second-level domain names.

Example	Possible applicants	Potential risks
tobacco.health	Tobacco manufacturers, industry marketing representatives, questionable corporate social responsibility platforms	Misinformation regarding health risks associated with tobacco use and products. Use of economic incentives and unregulated online marketing (eg, cigarette coupons) to induce demand for products.
vaccination.health	Anti-vaccination activists, vaccination adverse event plaintiff attorneys/solicitors, faith-based groups opposed to vaccinations on non-scientific grounds	Misinformation regarding the health risks associated with vaccination use could lead to public misperception and fear, resulting in lower vaccination rates and potential impact on maintaining population herd-immunity.
diet.health	Obesity-related food and beverage manufacturers, marketing companies of “health” products and related weight loss supplements without proven efficacy, direct-to-consumer advertising by pharmaceutical manufacturers.	Misinformation regarding health behavior and risks associated with obesity could result in unhealthy consumption behavior, promotion of unhealthy foods and beverages, use of unapproved/non-scientifically validated weight loss products, and possible overprescribing of obesity-related drugs through DTCA.
miraclecure.health	Telemarketers with unproven medical and health products, marketers of unapproved treatments (eg, unregulated stem cell clinics), marketing towards vulnerable patient populations (eg, rare diseases, diseases without treatment options)	The claim of this second-level domain name alone is cause for concern as it implies a “miracle” cure for a certain health-related condition. Whether such clearly risky descriptive domains will be restricted or reserved by current .health applicants is not clear.

### .health Domain Controversy

Reflecting some of the above concerns, in January 2013, the International Medical Informatics Association (IMIA) filed an objection based on community opposition to the four .health gTLD applicants [15]. IMIA stated that all failed to demonstrate how their use of .health would be in the public interest, none had adequate consumer protections, and all were solely commercial in nature without any representation from the health community. Other stakeholders, including France, Mali, WHO, Save the Children, the HealthOntheNetFoundation, other nongovernmental organizations (NGOs), as well as the European Commission, voiced similar concerns [16,17].

In March 2013, following these objections, ICANN’s Independent Objector (IO), an impartial party acting in the public interest, lodged formal objections to all four .health applicants [18]. Additionally, ICANN’s At-Large Advisory Committee, which vets objections to gTLD applications, similarly voted to support an objection for three of the four applicants (except Afilias; the provider licensure provision may have been a differentiating factor) [19]. However, in a subsequent decision, the International Chamber of Commerce (ICC), an entity reviewing disputes filed by the IO, denied *all* limited public interest objections filed against current active .health applicants (ie, Donuts Inc, Affilias, DotHealth LLC) effectively clearing the way for future procedural decisions, favoring award of .health most likely through a bidding system [20,21]. Interestingly, objections filed by the IO for other health-related gTLDs (eg, .hospital, .med) described below and in Table 3 have been upheld. Yet ICANN has not explained how these gTLDs are more important than .health, thus creating a great deal of inconsistency in the objection process.

Another concerning development occurred in October 2013, when ICANN’s New gTLD Program Committee issued a separate decision to re-categorize .health as being appropriate for open entry, effectively exempting it from certain “Safeguard Advice” (including requiring regulatory body oversight/licensure) that would limit its use [19]. In this decision, ICANN prioritized implementation of safeguards for “highly-regulated” gTLDs such as .sucks, .wtf, .poker, .lawyer, and .bank, over its limited safeguards proposed for .health [19].

### Other Health-Related gTLD Applicants

Controversy regarding the three remaining applicants for .health has sparked global debate on eHealth governance and concerns from various public health stakeholders [1]. However, other important health-related gTLDs have not been adequately analyzed nor discussed that face similar concerns. In addition to reviewing current applicants for .health, other health-related gTLDs with key terms including .care, .clinic, .dental, .dentist, .diet, .doctor, .healthcare, .hospital, .medical, and .surgery are in play (Table 3). Alarmingly, Donuts Inc-related subsidiaries are named as applicants for all of the above health-related gTLD strings, and similarly will not require tangible restrictions or verification of licensure/credentials for future registrants. Only two other applicants for the .doctor gTLD have any verification systems, such as mandated medical licensure, prior to .doctor domain use.

Large multinational pharmaceutical companies are also active in gTLD applications. This includes applications by 6 pharmaceutical manufacturers including Pfizer, Eli Lilly and Company, Merck, Sanofi, Hisamitsu Pharmaceutical, and Teva Pharmaceutical Industries. Largely these corporations are registering for brand and trademark protection purposes and generally limit registrants to company affiliates or licensees/authorized partners, which may represent a legitimate use of a gTLD. One application for the proprietary name of erectile dysfunction drug Cialis by Eli Lilly was pursued but is no longer active. However, direct-to-consumer advertising of prescription products is not allowed in the vast majority of countries other than the United States and New Zealand, and it may be unlawful for pharmaceutical manufacturers to engage in multijurisdictional online advertising that could occur through future gTLDs [7,22].

In contrast to the .health gTLD applicants, the .med gTLD (abbreviation for medical) have included partnership with more reputable health care stakeholders and information technology providers and have processes for restricting future registrants. This included the Cleveland Clinic’s engagement with Medistry LLC for its .med application requiring a request for proposal to vet qualifications of future registrants and Google Inc’s own application (Charleston Road Registry Inc). However, despite the Cleveland Clinic affiliation representing a higher level of legitimacy compared to .health applicant counterparts, community objections to its .med applications were upheld by the ICC (along with objections to Google Inc’s application), whereas similar objections raised by the IO for .health have been denied.

**Table 3:** Other health-related gTLD applicants.

gTLD string	Description of gTLD	Applicant name	Application status and country	Applicant type	Specific restrictions
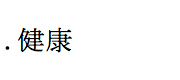	Chinese characters for the term “healthy” (jiankang)	Stable Tone Limited	Passed ICANN Initial Assessment Hong Kong	Limited Company	Agrees to mitigate against sites that sell counterfeit pharmaceuticals or other violating products/services
.cancerresearch	gTLD string for Cancerresearch	Australian Cancer Research Foundation (ACRF)	Passed ICANN Initial Assessment Australia	Australian public company limited by guarantee, nonprofit company, charitable institution	Registration of .cancerresearch domain names will be restricted to entities affiliated with ACRF
.care	Descriptive term in health	Goose Cross (Donuts Inc)	Passed ICANN Initial Assessment USA	Limited liability company	No specific restrictions
.clinic	Health care location/site	Goose Park, LLC (Donuts Inc)	Passed ICANN Initial Assessment USA	Limited liability company	No specific restrictions
.diet	Health behavior associated with weight	dot Diet Limited	Passed ICANN Initial Assessment Gibraltar	Limited liability company	Governing Council as advisory board. Protection of Intergovernmental Organization names; no other specific restrictions
	Health behavior associated with weight	Uniregistry, Corp.	Passed ICANN Initial Assessment Cayman Islands	Exempted corporation	No specific restrictions
	Health behavior associated with weight	Pioneer Hill, LLC (Donuts Inc)	Passed ICANN Initial Assessment USA	Limited liability company	No specific restrictions
.dds	Abbreviation for dental profession licensure	Top Level Domain Holdings Limited	Passed ICANN Initial Assessment British Virgin Islands	Publicly traded corporation	Open to licensed dentist (Doctor of Dental Surgery degree recognized by accrediting body) only
	Abbreviation for dental profession licensure	Charleston Road Registry Inc	Passed ICANN Initial Assessment USA	Corporation (Google parent company)	Verification of dentist or dental practices through membership with American Dental Association
.dental	Descriptive term for oral health practice	Tin Birch, LLC (Donuts Inc)	Passed ICANN Initial Assessment USA	Limited liability company	No specific restrictions
.dentist	Descriptive term for dental health care professional	Outer Lake, LLC (Donuts Inc)	Passed ICANN Initial Assessment USA	Limited liability company	No specific restrictions
.doctor	Descriptive term for physician health care professional	DotMedico TLD Inc	Passed ICANN Initial Assessment Republic of Seychelles	International business company	Registrants of .doctor will have to participate in a verification of their medical license as confirmed through data issued by the issuing authority of that license
		Brice Trail, LLC (Donuts Inc)	Passed ICANN Initial Assessment USA	Limited liability company	No specific restrictions
		The Medical Registry Limited	Passed ICANN Initial Assessment USA	Corporation	All entities registering within the .doctor TLD being required to produce verifiable credentials linked to evidence of professional qualifications or affiliation
.healthcare	General descriptive term	Silver Glen, LLC (Donuts Inc)	Passed ICANN Initial Assessment USA	Limited liability company	No specific restrictions
.hiv	Descriptive term for human immunodeficiency virus infectious disease	dotHIV gemeinnuetziger e.V.	Passed ICANN Initial Assessment Germany	Gemeinnuetziger eingetragener Verein under German law (German charitable Incorporated nonprofit Association)	Open to all registrants. Aims to distribute at least 50% of all registration fees towards fundraising for HIV/AIDS.
.hospital	General term for health care facility	Ruby Pike, LLC (Donuts Inc)	Passed ICANN Initial Assessment; IO objection sustained USA	Limited liability company	No specific restrictions
.med	Abbreviated term for “medical”	Medistry LLC	Passed ICANN Initial Assessment; IO objection sustained USA	Limited liability company	Partnership to operate and maintain the gTLD with The Cleveland Clinic; will use request for proposals process to vet qualification of registrants
	Abbreviated term for “medical”	HEXAP SAS	Passed ICANN Initial Assessment France	SAS company	Eligibility restricted to a list of licensed practitioner and health care entities with re-verification upon domain name renewal
	Abbreviated term for “medical”	Charleston Road Registry Inc (Google Inc)	Passed ICANN Initial Assessment; IO objection sustained USA	Corporation	Limiting registration to only verified doctors
.medical	General descriptive health term	Steel Hill, LLC (Donuts Inc)	Passed ICANN Initial Assessment USA	Limited liability company	No specific restrictions
.pharmacy	General term for health care facility	National Association of Boards of Pharmacy	Passed ICANN Initial Assessment In Contracting USA	Nonprofit institution	Goal of forming .pharmacy gTLD as an international safe namespace for legitimate online pharmacies. Applicants will be vetted for applicable regulatory standards, pharmacy licensure, drug authenticity, and valid prescription requirements
.rehab	General term for medical treatment	United TLD Holdco Ltd.	Passed ICANN Initial Assessment Cayman Islands	Corporation	No specific restrictions
.surgery	General term for health care procedure	Tin Avenue, LLC (Donuts Inc)	Passed ICANN Initial Assessment USA	Limited liability company	No specific restrictions

Similar to health care licensure verification requirements, the Australian Cancer Research Foundation (.cancerresearch) has policies requiring future registrants to be entities affiliated with the Foundation. The American Heart Association filed for two gTLDs (.heart and .stroke) but has subsequently withdrawn these applications (Table 4). In addition, the nonprofit German entity, dotHIV gemeinnuetziger e.V., applied for a .hiv gTLD to specifically leverage health communication and fund raising [23]. It has no restrictions on use and claims it will distribute at least 50% of future registration fees towards fundraising for the disease.

Some gTLD professional medical organization applicants have also initiated safeguards to protect public health, including the US National Association of Boards of Pharmacy (NABP) who is the sole applicant for .pharmacy and is currently in the award/contracting process. NABP’s application includes the stated goal of addressing the growing problem of illicit online pharmacies. In its application, it commits to establishing a safe and reliable international online space for online pharmacies requiring vetting of applicants for applicable regulatory standards, licensure, drug authenticity, and valid prescription requirements.

**Table 4:** Examples of withdrawn applications.

gTLD string	Description of gTLD	Applicant name	Application status and country	Applicant type	Specific restrictions
.heart	General descriptive term/organ	American Heart Association, Inc	WithdrawnUSA	Not-profit institution	Various criteria as a restricted name space for registrants including specific requirements for hospitals, universities, individuals, and corporations.
.med	Abbreviated term for “medical”	DocCheck AG	WithdrawnGermany	Publicly traded corporation	Proof of formal or long-standing training and/or experience in any field of medicine. Target group for field of medicine very broad.
.stroke	General term for medical condition	American Heart Association, Inc	WithdrawnUSA	Not-profit institution	.STROKE will be a single registrant TLD and will have only registrations that are specific to and managed by AHA directly

## Discussion

The review and delay of the .health gTLD and pending status of other health-related gTLDs provides the global community an opportunity to shape this important component of future eHealth governance. Clearly, use of the Internet for health information and its impact on health behavior is now a key issue in global health. In response, the global health community should recognize the importance of establishing a dedicated, safe, reliable, trustworthy, and accessible space for health information on the Internet to ensure that public health needs are met appropriately.

Establishing an evidence-based and appropriately validated .health gTLD can promote this goal and could be accomplished by international multistakeholder participation. Although WHO rightly expressed interest in a .health domain over a decade ago, a serious challenge to regain governance and oversight over .health has not occurred. This is despite the 66^th^ World Health Assembly (WHA) calling for all health-related gTLDs to be used to promote public health. The WHA also urged its member states and the director-general to work within ICANN’s Governmental Advisory Committee to ensure proper governance and operation of all health-related gTLDs—specifically .health [24]. However, at present, there is a lack of immediate and tangible action to intercede in the ongoing ICANN approval process, which is rapidly moving towards a final conclusion.

Global public health stakeholders should demand collective action by WHO, health-related United Nations organizations, multilateral/bilateral health agencies, national governments, NGOs, medical/patient professional societies, civil society, and other public health stakeholders. All should commit to securing a safe space for the health Internet that abides by ethical principles, practices, and rules that honor public health interests first and foremost. This community should call for ICANN to treat .health and other health-related gTLDs as protected, differentiating them from the other gTLD applications given their potential social and health impacts [1]. This should include reinforcing the universally agreed upon concept argued by the IO to the ICC that health is not just any commodity and that under international law, “health” is recognized as a fundamental human right, which includes the right to access accurate health information. Unfortunately, the ICC has rejected this position and the concerns of various public health stakeholders in its review of .health objections.

In response, concrete action should begin with calling for an immediate moratorium and suspension on ICANN decisions on .health and health-related gTLD applications to provide the global health community necessary time to explore an efficient, safe, and equitable governance structure that prioritizes stakeholder participation with the shared goal of ensuring adequate privacy, ethical use, and ensuring trust and protection of online health consumers. Indeed, WHO had specifically requested a postponement in a letter to ICANN in April 2012, though its actions and recent WHA resolution have been interpreted by the ICC as not definitive enough to support a call for protection of .health [19].

Future eHealth governance approaches to ensure the appropriate management of .health could be accomplished by requesting ICANN to re-categorize .health as a sponsored gTLD and proactively appoint WHO its sponsor [1]. By re-categorizing .health (similar to eligibility requirements in place since 2001 for .edu as a sponsored gTLD), WHO would develop policies to ensure accountability and transparency in gTLD operations that meet the best interests of the global health community and enforce eligibility rules regarding all future .health registrants [1].

However, in order to ensure a truly inclusionary and multistakeholder process necessary for the equitable management of .health, WHO’s possible appointment as gTLD sponsor should be governed by a diverse and globally representative board of global health stakeholders in partnership with responsible Internet service providers. This governance mechanism can have representation and be organized into subject-specific advisory panels to review and recommend content to be included on for .health. It can also agree to standards of quality online health information and work towards developing globally accepted norms and standards for content (eg, evidenced-based information, public health agency information). This should also include health care providers appropriately vetted for content review, licensure, credentialing, and other quality indicia to ensure legal marketing of health-related products/services within appropriate jurisdictions.

At a minimum, the international community should demand a postponement of any imminent ICANN decisions on current .health applications and other health-related gTLDs reviewed above. This is particularly important for those health-related gTLDs that are currently being aggressively pursued by Donuts Inc as the sole applicant. Instead, multistakeholder groups with transparent and accountable governance mechanisms and a mandate to promote public health are key to ensuring the trust and credibility of health on the Internet.

## Conclusion

The importance of establishing an inclusive yet reliable presence for health information online is critical to future global health outcomes given the growing importance of the health Internet. However, .health and many other health-related gTLDs are now on sale to private sector entities that largely permit open and unrestricted use. Yet, the globalized nature of the Internet, the public health need for privacy, security, and quality health information, and the rapid expansion of online health technologies demonstrate a critical need to ensure proper governance of future health domains. Focusing on the public good can be a first and crucial step to ensure an accurate, reliable, and evidence-based online presence for health for this generation and the next.

## Abbreviations

ACRF: Australian Cancer Research Foundation
AHA: American Health Association
gTLD: generic top-level-domain
ICANN: The Internet Corporation for Assigned Names and Numbers
ICC: the International Chamber of Commerce
IGO: intergovernmental organization
IMIA: the International Medical Informatics Association
IO: Independent Objector
NAPB: National Association of Boards of Pharmacy
NGO: nongovernmental organization
TLD: top-level-domain
WHA: World Health Assembly
WHO: World Health Organization
